# A MEMS-Based Quad-Wavelength Hybrid Plasmonic–Pyroelectric Infrared Detector

**DOI:** 10.3390/mi10060413

**Published:** 2019-06-21

**Authors:** Anh Tung Doan, Takahiro Yokoyama, Thang Duy Dao, Satoshi Ishii, Akihiko Ohi, Toshihide Nabatame, Yoshiki Wada, Shigenao Maruyama, Tadaaki Nagao

**Affiliations:** 1 International Center for Materials Nanoarchitectonics, National Institute for Materials Science, 1-1 Namiki, Tsukuba 305-0044, Japan; doan.tunganh@nims.go.jp (A.T.D.); yokoyamatakahiro1007@gmail.com (T.Y.); dao.duythang@nims.go.jp (T.D.D.); sishii@nims.go.jp (S.I.); ohi.akihiko@nims.go.jp (A.O.); nabatame.toshihide@nims.go.jp (T.N.); 2 Department of Condensed Matter Physics, Graduate School of Science, Hokkaido University, Kita-10 Nishi-8 Kita-ku, Sapporo 060-0810, Japan; 3 Research Center for Functional Materials, National Institute for Materials Science, 1-1 Namiki, Tsukuba 305-0044, Japan; wada.yoshiki@nims.go.jp; 4 National Institute of Technology, Hachinohe College, 16-1 Uwanotai, Tamonoki, Hachinohe City, Aomori Prefecture 039-1192, Japan; maruyama@ifs.tohoku.ac.jp

**Keywords:** infrared detector, quad-wavelength, hybrid plasmonic–pyroelectric, MEMS-based, spectral selectivity

## Abstract

Spectrally selective detection is of crucial importance for diverse modern spectroscopic applications such as multi-wavelength pyrometry, non-dispersive infrared gas sensing, biomedical analysis, flame detection, and thermal imaging. This paper reports a quad-wavelength hybrid plasmonic–pyroelectric detector that exhibited spectrally selective infrared detection at four wavelengths—3.3, 3.7, 4.1, and 4.5 μm. The narrowband detection was achieved by coupling the incident infrared light to the resonant modes of the four different plasmonic perfect absorbers based on Al-disk-array placed on a Al_2_O_3_–Al bilayer. These absorbers were directly integrated on top of a zinc oxide thin film functioning as a pyroelectric transducer. The device was fabricated using micro-electromechanical system (MEMS) technology to optimize the spectral responsivity. The proposed detector operated at room temperature and exhibited a responsivity of approximately 100–140 mV/W with a full width at half maximum of about 0.9–1.2 μm. The wavelength tunability, high spectral resolution, compactness and robust MEMS-based platform of the hybrid device demonstrated a great advantage over conventional photodetectors with bandpass filters, and exhibited impressive possibilities for miniature multi-wavelength spectroscopic devices.

## 1. Introduction

Multispectral selectivity is of crucial importance in the development of modern infrared (IR) detectors for modern spectroscopic applications including multi-wavelength pyrometry [1,2,3,4,5], non-dispersive infrared (NDIR) gas sensing [6,7,8,9,10], biomedical analysis [11,12,13,14,15,16,17,18], flame detection [19,20,21,22], and thermal imaging [23,24,25]. Spectrally selective IR detectors that are based on resonant cavity enhanced (RCE) photodetectors exhibit excellent spectral sensitivity and fast responses [26,27,28,29,30,31]. However, the requirement for cryogenic cooling makes them bulky, heavyweight, excessively costly, and complicated for some applications. Pyroelectric and thermopile detectors offer the advantages of being able to be operated at room temperature and of having wide spectral responses. Conventional spectrally selective uncooled detectors typically use passband filters mounted in front of the sensing element to filter out signals at the wavelengths that are out of interest, resulting in bulky designs and limited wavelength tunability.

Over the last two decades, the advent of plasmonic metamaterials, which are artificially structured materials with periodic subwavelength unit cells, has offered great freedom to tailor the absorption spectra [32,33,34,35,36]. The absorption peaks can be precisely controlled and manipulated by carefully designing the geometrical parameters of the unit cells. As the field of microelectromechanical systems (MEMS) has rapidly advanced, plasmonic perfect absorbers can be directly integrated on micromachined pyroelectric transducers to create compact, high-performance yet low-cost multi-wavelength detectors that operate at room temperatures. 

In this work, we proposed and implemented a quad-wavelength pyroelectric detector with four distinct plasmonic absorbers to selectively detect light in the mid-IR region. For NDIR multi-gas sensing applications, the four resonance wavelengths were determined at 3.3, 3.7, 4.1, and 4.5 μm, which corresponded to the centered absorption band of CH_4_, H_2_S, CO_2_, and N_2_O [37,38]. The spectral selectivity was achieved by the coupling of incident infrared light to resonant modes of Al-disk-array/Al_2_O_3_/Al perfect absorbers with various disk sizes. The top patterned resonators were hexagonal arrays of disks used to achieve wide-angle acceptance and polarization-insensitivity, which are highly desirable for many sensing applications. We chose Al as the plasmonic base metal because it is abundant on Earth and it is industry-compatible while still exhibiting low-loss plasmonic properties similar to noble metals such as Au, Ag in the IR region [39]. The model of the Al-disk-array/Al_2_O_3_/Al perfect absorber was first constructed in a computer-aided design (CAD) layout (Rsoft CAD, Synopsys’s Rsoft, Synopsys, Inc.) [40]. The absorptivities, electric field, and magnetic field distribution of the absorbers were simulated and optimized using the commercial rigorous coupled-wave analysis (RCWA) package and the FullWAVE package from Synopsys' Rsoft [40], which is a highly sophisticated tool for studying the interaction of light and photonic structures, including integrated wavelength-division multiplexing (WDM) devices [41,42], as well as nanophotonic devices such as metamaterial structures [34,43], and photonic crystals [44]. The sensing areas were designed as floating membranes above a void space to minimize thermal conduction, thereby improving the responsivity of the detector. The electromagnetic energy at the resonance wavelengths induced heat on the upper surface of the zinc oxide layer, which features pyroelectricity in thin film form. Due to the pyroelectric effect, a signal voltage was generated at the resonance wavelengths for each absorber. The on-chip design of the proposed quad-wavelength pyroelectric detector demonstrated the feasibility of integrating micro-detectors of different selective wavelengths into arrays with good CMOS compatibility. This opens the possibility of developing miniaturized and robust multi-color spectroscopic devices. 

## 2. Design and Fabrication 

### 2.1. Structure Design

**Figure 1:**
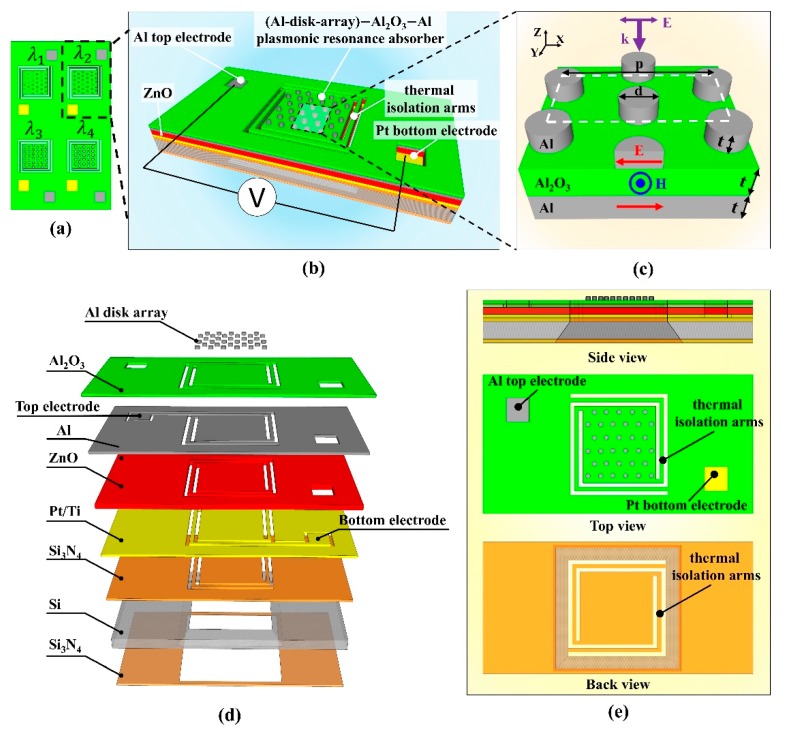
(**a**) Schematic illustration of the proposed quad-wavelength detector. (**b**) Illustration of the structural design of a single sensing element. (**c**) Illustration of the plasmonic perfect absorber with the indicated field lines at resonance (electric field E, red arrows; magnetic field H, blue arrows). (**d**) Exploded view of a single sensing element. (**e**) Side view, top view, and bottom view of a single sensing element.

The schematic diagram in Figure 1a illustrates the design layout of the proposed quad-wavelength detector. Four individual sensing elements were directly integrated on the same complementary metal-oxide-semiconductor (CMOS) platform with a size of 0.5 × 1.0 cm^2^ to selectively detect IR radiation at four resonant wavelengths of 3.3, 3.7, 4.1, and 4.5 μm. The structural design of a single sensing element is illustrated in Figure 1b. From the top to bottom, it consisted of an Al-disk-array/Al_2_O_3_/Alperfect absorber structure with an active area of 200 × 200 μm^2^, a 300 nm-thick pyroelectric zinc oxide thin film sandwiched between the Al back plate of the absorber and a 100 nm Pt/10 nm Ti bottom electrode, and a membrane-based CMOS substrate. A 300 nm-thick layer of silicon nitride was deposited on both sides of the silicon substrate to supply adequate mechanical strength for the membrane structure. The silicon wafer was 380 μm thick. The width and the length of the supporting arms were 20 μm and 400 μm. The cross-sectional profile of the unit cell is shown in Figure 1c. The Al-disk-array/Al_2_O_3_/Alperfect absorber consisted of an Al disk array as the resonator, an Al_2_O_3_ layer as the middle insulator, and an Al film as the back reflector. The thicknesses of the three layers were identical and were represented as t. The Al disks of diameter d were arranged in a periodic hexagonal array of periodicity p. After some numerically computational efforts based on the RCWA analysis, the optimized geometrical dimensions for the perfect absorbers were taken as p = 2.0 µm, t = 100 nm, and d = 0.97, 1.25, 1.35, 1.60 μm for the individual sensing elements at resonance wavelengths of 3.3, 3.7, 4.1, and 4.5 μm, respectively. When an incident IR radiation impinged on the top surface of the device, its external oscillating electric field induced electric dipoles inside the Al disks, which excited anti-parallel currents between the disk array and the back reflector. These circular currents produced a magnetic flux opposing the external magnetic field, resulting in a magnetic resonance. By adjusting the geometrical parameters of the Al disk-array, we could tailor the electromagnetic response of the structure to the external electromagnetic field to achieve selective absorption. 

The exploded view of a single sensing element is shown in Figure 1d. It was shown that the bottom layer of plasmonic metamaterial perfect absorber was also utilized as the top electrode of the pyroelectric detector. In this design, the zinc oxide layer was stacked below and in intimate contact with the plasmonic resonance absorber to fully exploit the spectrally selective radiation. It is also worth noting that we chose zinc oxide as the pyroelectric material due to its advantages of being nontoxic and compatible with the semiconductor process. This design is also applicable for other pyroelectric materials such as lead zirconate titanate, lithium tantalate, lithium niobate, barium strontium titanate, deuterated triglycine sulfate, and others. The side view, top view, and back view of the single sensing elements are shown in Figure 1e, indicating the micromachined floating membrane design of the sensing element.

### 2.2. Simulation

**Figure 2:**
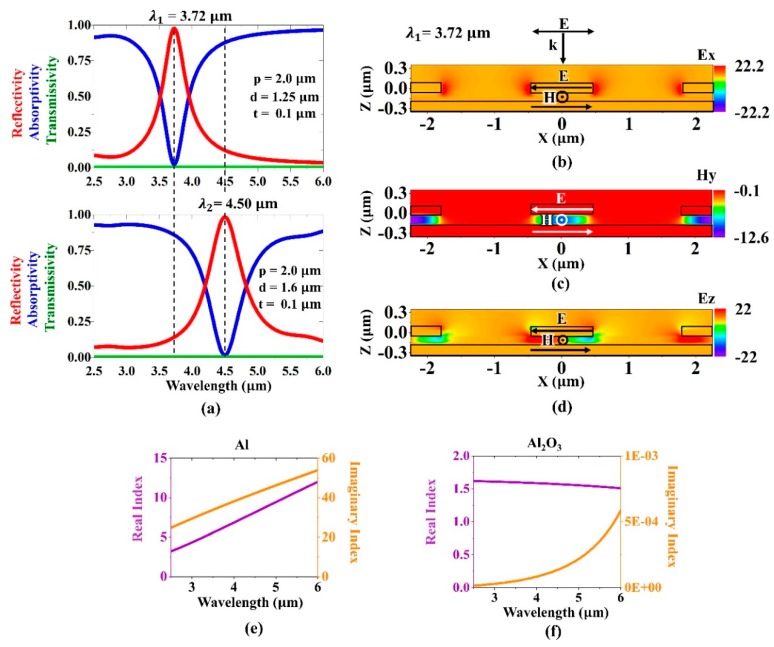
(**a**) Reflectivity, absorptivity, and transmissivity characteristics of the plasmonic perfect absorber at the resonance wavelengths of 3.7 μm and 4.50 μm. The simulated amplitude plots of (**b**) electric field in x direction, (**c**) magnetic field in y direction, (**d**) electric field in z direction at the resonance wavelength of 3.7 μm, illustrates the highly localized characteristics of the plasmonic perfect absorber. The refractive index dispersion data of (**e**) Al and (**f**) Al_2_O_3_ used in the simulations was taken from literature [45].

To tailor and optimize the spectrally selective absorption, the optical properties of the plasmonic absorbers were simulated by implementing rigorous coupled-wave analysis (RCWA) (DiffractMOD, Synopsys' Rsoft). Figure 2a shows the simulated reflectivity, transmissivity, and absorptivity characteristics of the two Al-disk-array/Al_2_O_3_/Al perfect absorbers which had diameters of 1.25 and 1.6 μm, respectively. As the optimal parameters, the unit cells had periodic dimensions of 2.0 μm, and the thicknesses of the Al disks, Al_2_O_3_ dielectric spacer and Al back reflector were all 100 nm. Since the continuous metal back reflector was thick enough to block light transmission, the transmissivity was zero across the investigated wavelength range. With disk diameters of 1.25 and 1.6 μm, the simulated absorptivities at normal incidence were resonantly enhanced up to 0.99 and 0.94 at the resonance wavelengths of 3.72 and 4.05 μm, with a full width at half maximum (FWHM) of 0.44 and 0.63 μm, respectively.

To gain insight into the resonant behavior of the Al-disk-array/Al_2_O_3_/Al perfect absorber, a full wave simulation based on the finite-difference time-domain (FDTD) method (FullWAVE, Synopsys' Rsoft) was performed at the resonance wavelength of 3.72 μm. The dielectric functions of Al, Al_2_O_3_ were taken from the literature. The simulated electric field distribution in x direction (Figure 2b) shows that the electric field was localized at the edges of the Al disks, indicating the induced electric dipoles. The magnetic field was intensively confined under the center of the top resonator (Figure 2c), for which the antiparallel currents were excited in the top Al disks and the bottom Al reflector (Figure 2d). 

**Figure 3:**
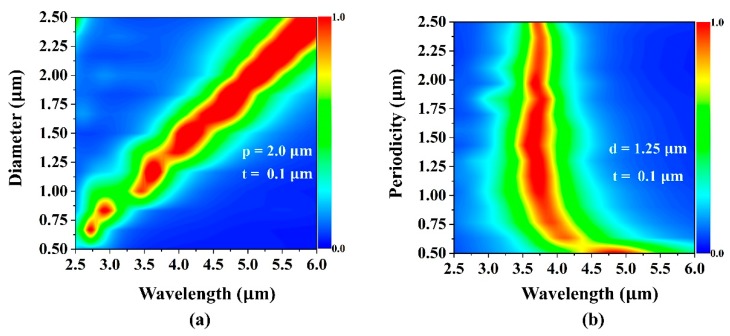
(**a**) The periodicity dependence with p changed from 0.5 to 2.5 μm while keeping the diameter and insulator thickness unchanged at 1.25 μm and 0.1 μm, respectively. (**b**) The diameter dependence with d changed from 0.5 to 2.5 μm, while keeping the periodicity and insulator thickness unchanged at 2.0 μm and 0.1 μm, respectively.

The dependence of the resonant modes of the plasmonic absorber as a function of disk size is illustrated in Figure 3a. Accordingly, the peak absorption was found to shift almost linearly with the disk diameter. Meanwhile, the resonance wavelength was not greatly affected by the variations in periodicity of the unit cells (Figure 3b). Due to this robust tunability with respect to the disk size, the electromagnetic response of these Al-disk-array/Al_2_O_3_/Al structures were engineered to achieve resonant absorption at multiple desired wavelengths.

**Figure 4:**
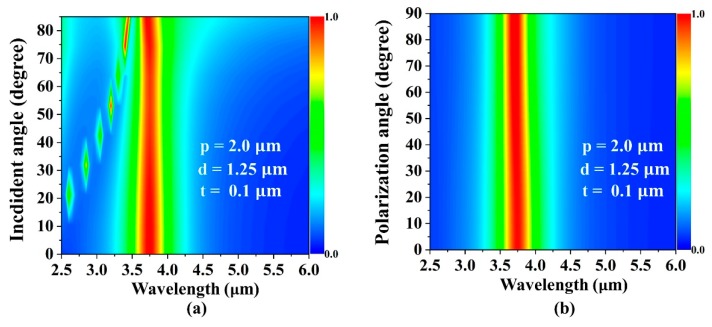
Simulated absorptivity spectrum as a function of (**a**) the incident angle and (**b**) polarization angle for an absorber with p = 2.0 μm, d = 1.25 μm, and t = 0.1 μm.

Figure 4a shows the simulated incident angle-dependent absorptivity with the geometrical parameters p = 2.0 μm, d = 1.25 μm, and t = 0.1 μm. The simulation results showed that the proposed plasmonic perfect absorber exhibited strong absorption independence to the incident angles. The absorption still remained near unity when the incident angle was increased up to 85°. Figure 4b shows the polarization-dependent absorptivity with the geometrical parameters p = 2.0 μm, d = 1.25 μm, and t = 0.1 μm. The result indicated that the resonances of the plasmonic absorber were almost unchanged when the polarization angle changed from 0 to 90°. The symmetrical design typically resulted in a wide-angle acceptance and polarization independence, which was desirable for most of the sensing applications.

### 2.3. Fabrication

**Figure 5:**
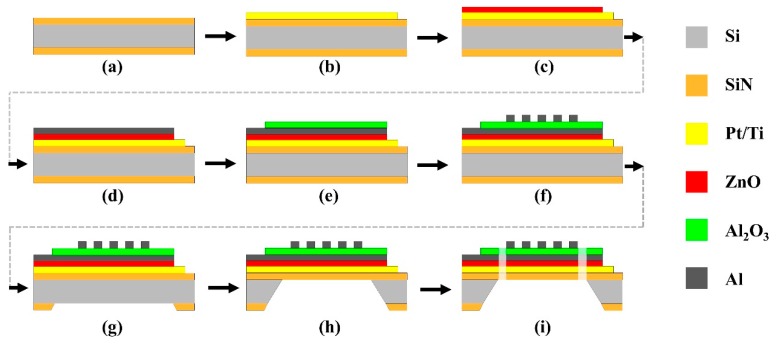
CMOS-compatible microfabrication process: (**a**) Rapid thermal oxidation of the silicon wafer and deposition of Si3N4 on both sides; (**b**) Deposition and lift-off of the Pt bottom electrode; (**c**) Deposition and lift-off of ZnO; (**d**) Deposition and lift-off of Al; (**e**) Deposition and lift-off of Al_2_O_3_; (**f**) E-beam lithography of the Al disk; (**g**) Dry etching backside of Si_3_N_4_; (**h**) Wet etching of Si by KOH; (**i**) Dry etching of Si_3_N_4_.

The fabrication of the proposed detector involved standard photolithography with dry and wet etching techniques. The microfabrication process of the MEMS-based hybrid plasmonic–pyroelectric detector is shown in Figure 5. The device was fabricated on commercial double-side polished *n*-type silicon prime wafers with <100> orientation in cleanroom facilities.

First, an 80 nm-thick SiO_2_ insulator was formed by the dry thermal oxidation of the silicon wafer. The fabrication process was followed by depositing a 300 nm-thick silicon nitride (Si_3_N_4_) layer onto both sides of the silicon substrate by radio frequency (RF) reactive sputtering using a boron-doped p-type Si target processed in ambient N_2_ as a reactive gas with a flow rate of 20 sccm (sputter i-Miller CFS-4EP-LL, Shibaura, Tokyo, Japan) (Figure 5a). This silicon nitride layer isolated the thermal conduction to the substrate, which could enhance the pyroelectric signal and the overall responsivity of the device. Then the silicon nitride layer was put into a rapid thermal annealing process at 1000 °C for 1 min. After that, laser direct writing lithography (μPG 101 Heidelberg Instruments) was performed to pattern the bottom electrode on top of the silicon nitride layer with a double layer of resists. The 100 nm Pt/10 nm Ti bottom electrodes were deposited in an electron beam evaporator at ambient temperatures, followed by a lift-off process in acetone (Figure 5b). Then, the sputter deposition of the 300 nm-thick zinc oxide layer was followed by laser direct writing lithography with positive photoresist combined with reactive-ion etching (RIE) using CF_4_ gases (Ulvac CE-300I) (Figure 5c). Next, the sputter deposition of the 100-nm-thick Al top electrode was followed by laser direct writing lithography with positive photoresist, and RIE using BCl_3_/Cl_2_ gases (Figure 5d). After exposing and developing the pattern, a 100 nm Al_2_O_3_ film was sputtered and then patterned by direct laser writing lithography combined with a RIE step using CF_4_ gas (Figure 5e). The 100-nm-thick Al disk array was patterned using the e-beam lithography and lift-off process (Figure 5f). The Si_3_N_4_ back layer serving as a RIE mask for the wet etching process of Si was patterned by RIE with CHF_3_ gas using a photoresist mask formed by direct laser writing lithography (Figure 5g). Then, the backside wet alkaline etching was implemented as follows (Figure 5h). A thin polymeric ProTEK^®^B3 protective coating was spun on the front side of the structure to provide a temporary wet-etch protection for the patterned Al-disk-array/Al_2_O_3_/Al resonator during alkaline etches. The Si layer from the back side of each sensor was completely released by anisotropic wet-etching by immersing the structure in a deep reactive KOH 40% aqueous solution, heating at 60 °C for 10 h. Then, the polymeric protective coating was removed by immersing it into acetone. Finally, reactive-ion etching of Si_3_N_4_ was carried out to form the supporting arms (Figure 5i). The Si_3_N_4_/ SiO_2_ composite layer was used as the membrane to support the zinc oxide pyroelectric film and absorber. Each sensing element was wire-bonded to the Cu electrodes attached on a glass plate. The materials and the fabrication process of the detector was fully compatible with the CMOS.

## 3. Results and Discussion

**Figure 6:**
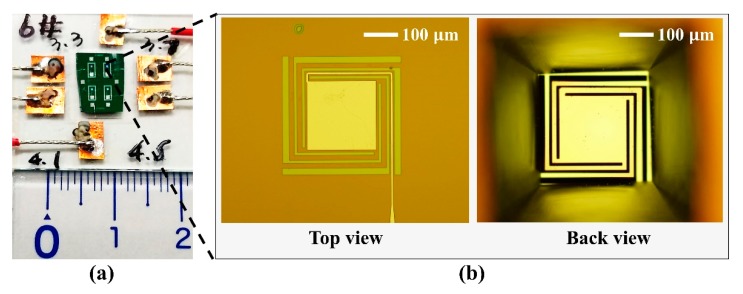
(**a**) Photo of a fabricated MEMS-based hybrid plasmonic–pyroelectric detector. (**b**) Microscope images of the top view and back view of the fabricated sensing element.

Figure 6a shows a photo of a fabricated MEMS-based hybrid plasmonic–pyroelectric detector, which had a width of 0.5 cm and length of 1 cm. It clearly demonstrates that multiple hybrid plasmonic–pyroelectric sensing elements were easily integrated on a standard CMOS platform to achieve multispectral selectivity without any additional bulky optical filters. Figure 6b shows the optical microscopy images of the top view and back view of a single sensing element with an active area of 200 × 200 μm. It verifies that the sensing area was suspended by the long thermal isolation arms. The suspended area remained flat without any additional stress-reducing process.

**Figure 7:**
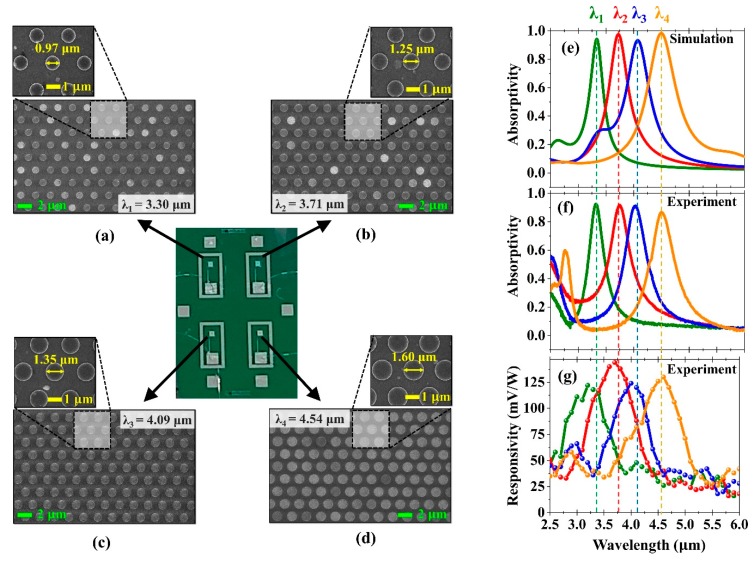
(**a**–**d**) Scanning electron microscope (SEM) images of the fabricated disk array patterns at four resonance wavelengths. (**e**,**f**) Simulated and measured absorptivities of the perfect absorbers at four resonance wavelengths. (**g**) Measured responsivity of the quad-wavelength detector.

While the thicknesses of the sputtered layers could be precisely controlled by establishing optimum sputtering conditions and deposition rates, the process of transferring the shape and size of the Al disk arrays pose further challenges due the complex multi-stage photolithography of the sub-micron patterning. The residual photoresist and non-uniformity may result in degradation from the expected performance. Figure 7a–d show the scanning electron microscope (SEM) images of the hexagonal arrays of the Al disk resonators with diameters of 0.97, 1.25, 1.35, 1.60 μm. It was shown that the Al disk arrays fabricated on top of the membrane structures were well-defined and homogeneously distributed, indicating that the patterning process was precisely implemented. Because the Al disk arrays were patterned using electron-beam lithography with sub-10 nm resolution, there was a tolerance of a few nm in the diameter of the disks.

The reflectance spectrum of each sensing element was measured using a Fourier transform infrared spectrometer (FTIR) (Thermo Scientific Nicolet iS50, Thermo Fisher Scientific, Waltham, MA, USA), coupled with a microscope (Nicolet Continuum FTIR Microscope, Thermo Nicolet). The reflectance spectra were normalized with respect to the reflectance from a gold film. Given that the transmittance was zero for the thick back reflector, the absorptivity spectra were calculated using the formula A = 1 − R, where A was the absorptivity, R was the reflectivity. The simulated and measured absorptivity spectra are shown in Figure 7e,f. The reflectance spectra were normalized with respect to the reflectance from a gold film. The four sensing elements exhibited absorptivity peaks of 0.92, 0.93, 0.85, and 0.87 at 3.32, 3.74, 4.06, and 4.51 μm, respectively, which were highly consistent with the corresponding simulated absorptivity peaks of 0.94, 0.99, 0.94, 0.98 at 3.33, 3.72, 4.05, and 4.50 μm, respectively. The precise pattern transfer in the fabrication process resulted in small shifts of only a few tens nm and slightly lower magnitudes of absorption peaks. The FWHMs of the measured absorptivity curves were 0.35, 0.45, 0.49, 0.68 μm, compared to those of the simulated curves, which were 0.30, 0.44, 0.47, 0.63 μm. Together with the defined patterns and uniformity observed in the SEM images, the excellent agreement between the simulated and experimentally measured absorptivity proved the quality of the optimized fabrication method. 

The performance of the detector was evaluated by measuring its spectral responses to the IR radiation from a wavelength-tunable pulsed laser system in the range of 2.5–6.0 μm. The pulse width of the laser was 104 fs, and the repetition rate was 1 kHz. The IR laser beam was guided to the sensing area of the detector. The output electrical signal was amplified with a preamplifier and demodulated with a lock-in amplifier. The spectrally selective absorption of laser pulses at resonant wavelengths was due to the excitation of highly localized magnetic and electric dipole resonances, which was evidenced by the simulated field distributions (see Figure 2b–d). Such strong resonances efficiently confined the electromagnetic energy and provided sufficient time to convert it into resistive heat within the Al disks and the continuous Al back plate. Al_2_O_3_ underwent almost no loss in the mid-infrared region (see Figure 2f); that is, dielectric losses in the spacer layer were negligible in contrast to the plasmonic perfect absorbers operating in lower frequencies, such as in the microwave and terahertz region, in which the absorption was mainly due to dielectric losses. The heat at the continuous Al back plate that was associated with spectrally selective absorption was directly transferred to the zinc oxide thin film, which was among the materials that were electrically polarized due to their c-axis-oriented textured crystal structure [46,47,48]. The zinc oxide film consequently became electrically polarized with changes in the temperature, and the amount of electric charges was also proportional to the temperature fluctuation. The electrically polarized zinc oxide film hence produced a voltage between the Al and Pt electrodes. Pt was chosen as the bottom electrode because it has been reported that the hexagonal arrangement of the atoms in the (111) plane of Pt facilitated the nucleation and growth of hexagonal zinc oxide oriented along the [0001] direction [49]. In Figure 7g, the measured spectral responsivity curves of the four sensing elements are plotted. As seen from the results, the spectral responses of the four sensing elements were 125, 150, 126, 128 mV/W at 3.30, 3.71, 4.09, and 4.54 μm, respectively. The excellent measured spectral responses proved that the detector exhibited low thermal drift and thermal noise due to the low mass of the membrane-based design. The measured FWHMs of the spectral responses were 0.94, 1.02, 1.10, 1.20 μm, respectively, which were approximately two times broader than those of the absorptivity measured using the FTIR spectrometer. The broadened spectra could be partly attributed to the spectral bandwidth of the IR pulsed laser and the fluctuation of the incident angles and positions of the laser pulses. These experimental results clearly demonstrated that the proposed multispectral hybrid plasmonic–pyroelectric detectors can be fabricated and easily integrated into existing IR spectroscopic devices such as miniature NDIR sensors, chemical- and bio-sensors, photoacoustic imaging systems, and thermographic cameras. As an example, on-chip multi-spectral infrared detectors may offer an opportunity to develop miniaturized and robust photoacoustic computed tomography [50,51]. Specifically, in all-optical photoacoustic imaging systems, shifts in the wavelengths of light are able to be detected and analyzed using an array of multi-spectral micro-detectors.

## 4. Conclusions

We proposed and successfully fabricated and characterized a MEMS-based quad-wavelength hybrid plasmonic–pyroelectric infrared detector. Our device exploited the great tunability and high spectral resolution of patterned Al-disk-array/Al_2_O_3_/Al resonant perfect absorbers together with the MEMS-based pyroelectric platform. The absorption spectra of the plasmonic perfect absorbers were simulated and optimized using the FDTD and RCWA methods. The fabricated device demonstrated the spectrally selective detection of IR radiation at 3.3, 3.7, 4.1, and 4.5 μm, which are in the atmospheric window, with FWHMs of 0.94, 1.02, 1.10, 1.20 μm and responsivities of 125, 150, 126, 128 mV/W, respectively. These results demonstrate the feasibility and great potential of multispectral hybrid plasmonic–pyroelectric infrared detectors for a wide range of modern IR spectroscopic devices.
